# Adverse Drug Event Prediction Using Noisy Literature-Derived Knowledge Graphs: Algorithm Development and Validation

**DOI:** 10.2196/32730

**Published:** 2021-10-25

**Authors:** Soham Dasgupta, Aishwarya Jayagopal, Abel Lim Jun Hong, Ragunathan Mariappan, Vaibhav Rajan

**Affiliations:** 1 Mallya Aditi International School Bangalore India; 2 School of Computing National University of Singapore Singapore Singapore; 3 Department of Information Systems and Analytics National University of Singapore Singapore Singapore

**Keywords:** adverse drug event, knowledge graph, Embedding of Semantic Predications, biomedical literature

## Abstract

**Background:**

Adverse drug events (ADEs) are unintended side effects of drugs that cause substantial clinical and economic burdens globally. Not all ADEs are discovered during clinical trials; therefore, postmarketing surveillance, called pharmacovigilance, is routinely conducted to find unknown ADEs. A wealth of information, which facilitates ADE discovery, lies in the growing body of biomedical literature. Knowledge graphs (KGs) encode information from the literature, where the vertices and the edges represent clinical concepts and their relations, respectively. The scale and unstructured form of the literature necessitates the use of natural language processing (NLP) to automatically create such KGs. Previous studies have demonstrated the utility of such literature-derived KGs in ADE prediction. Through unsupervised learning of the representations (features) of clinical concepts from the KG, which are used in machine learning models, state-of-the-art results for ADE prediction were obtained on benchmark data sets.

**Objective:**

Due to the use of NLP to infer literature-derived KGs, there is *noise* in the form of false positive (erroneous) and false negative (absent) nodes and edges. Previous representation learning methods do not account for such inaccuracies in the graph. NLP algorithms can quantify the confidence in their inference of extracted concepts and relations from the literature. Our hypothesis, which motivates this work, is that by using such confidence scores during representation learning, the learned embeddings would yield better features for ADE prediction models.

**Methods:**

We developed methods to use these confidence scores on two well-known representation learning methods—DeepWalk and Translating Embeddings for Modeling Multi-relational Data (TransE)—to develop their *weighted* versions: Weighted DeepWalk and Weighted TransE. These methods were used to learn representations from a large literature-derived KG, the Semantic MEDLINE Database, which contains more than 93 million clinical relations. They were compared with Embedding of Semantic Predications, which, to our knowledge, is the best reported representation learning method using the Semantic MEDLINE Database with state-of-the-art results for ADE prediction. Representations learned from different methods were used (separately) as features of drugs and diseases to build classification models for ADE prediction using benchmark data sets. The methods were compared rigorously over multiple cross-validation settings.

**Results:**

The *weighted* versions we designed were able to learn representations that yielded more accurate predictive models than the corresponding unweighted versions of both DeepWalk and TransE, as well as Embedding of Semantic Predications, in our experiments. There were performance improvements of up to 5.75% in the F_1_-score and 8.4% in the area under the receiver operating characteristic curve value, thus advancing the state of the art in ADE prediction from literature-derived KGs.

**Conclusions:**

Our classification models can be used to aid pharmacovigilance teams in detecting potentially new ADEs. Our experiments demonstrate the importance of modeling inaccuracies in the inferred KGs for representation learning.

## Introduction

### The Challenge of Detecting Adverse Drug Events

Adverse drug events (ADEs) are unintended side effects of drugs that often lead to emergency visits, prolonged hospital stays, and worse patient outcomes [1]. They pose substantial clinical and economic burden—in the United States alone, morbidity and mortality costs associated with ADEs were estimated to be approximately US $528 billion in 2016 [2], and 1 in 3 drugs approved in the period from 2000 to 2010 had safety-related issues after release, some of which led to their withdrawal from the market [3].

Patients may be prescribed multiple drugs together when they have multiple coexisting ailments or for combination therapies, for example, in cancer [4]. In such cases, it is also possible for ADEs to occur because of a combination of drugs, also termed polypharmacy. Polypharmacy poses a higher risk of ADEs because of drug-drug interactions [5,6]. Polypharmacy is also an increasing burden to health care; estimates suggest that they cause nearly 74,000 emergency room visits and 195,000 hospitalizations annually in the United States [7].

In general, detecting ADEs is a challenging problem. Clinical trials are limited by the number and characteristics of patients tested as well as the duration of the observation period, and they may not detect all ADEs, especially those with long latency or those that affect only certain patient groups [8]. Detecting polypharmacy ADEs is even harder—although it is possible to test for a few drug interactions [9], it is computationally infeasible to test for all possible drug combinations [10]. Postmarketing drug safety surveillance, called pharmacovigilance, is routinely conducted to continuously update our knowledge of potential ADEs.

Spontaneous reporting systems, which collect voluntary reports of ADEs, have been the primary data source for pharmacovigilance. Mining these databases presents several challenges because of inherent reporting bias and incompleteness. Methods to detect ADE signals from other data sources such as social media and clinical data are being actively developed, but problems of quality and reliability limit the utility of these sources; the study by Ventola [1] provides a detailed survey. Biomedical literature, which forms another source of ADE signals, is also consulted during ADE mining from other sources. An advantage of these data over others is the presence of information relevant to potential causal assessment in the studies described. Furthermore, as biomedical knowledge grows, this source continues to expand and update itself systematically.

However, the scale is both an advantage and a hurdle. MEDLINE, the largest index of medical literature, contains more than 24 million articles, with more than a million new articles published annually [11]. This enormous scale makes it challenging to mine the data; therefore, to facilitate knowledge discovery from such unstructured data, standardized vocabularies and ontologies have been created. Furthermore, natural language processing (NLP) techniques have been developed to automatically infer both clinical concepts and their relations found in the literature. Such ontologies are also massive and continue to evolve with growing biomedical literature. An ontology can be viewed as a heterogeneous knowledge graph (KG) comprising multiple kinds of vertices (clinical concepts, eg, drugs and diseases) and edges (relations, eg, Treats and Is-A-Side-Effect).

Previous literature-based knowledge discovery systems—for ADE detection as well as for other applications—have mainly used text and graph mining or supervised learning methods [12-14] that require careful design of the features from text or graphs. For instance, certain patterns of relations (edges) among clinical concepts (vertices) may be used to mine ontologies for a potential ADE. Finding the right set of patterns can be challenging—for a given pair of concepts, evidence of an association, or lack thereof, cannot be discerned from the presence or absence of a single edge: two clinical concepts may be indirectly connected and, by multiple paths, be composed of several relations. Such manual feature engineering is cumbersome, time consuming, and does not scale with the rapidly evolving literature and literature-derived KGs.

**Figure 1:**
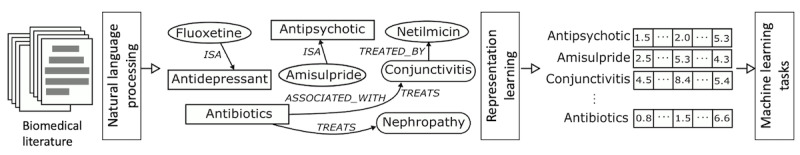
Knowledge graphs are obtained through natural language processing on biomedical literature. Clinical concept representations learnt from such graphs are used as features for machine learning tasks. ISA: is a.

To enable reasoning on such large and complex KGs and to automate feature engineering, most recent approaches use *graph embeddings* that encode the global structural properties of a given graph into vectorial *representations* of its vertices. With such representations, relations among clinical concepts can be computed algebraically using vectorial measures of similarity. Furthermore, these representations can be used as features directly in machine learning models for tasks such as association prediction or cluster detection (see Figure 1 for a schematic). Such approaches have yielded state-of-the-art results in many tasks, including ADE prediction from KGs [15].

Most representation learning methods have been designed for graphs from the internet, for example, social media or e-commerce, where the graph itself is assumed to have very few or no errors. In contrast, errors are common in literature-derived biomedical KGs because of the large and complex clinical vocabulary, which often contains inconsistently used abbreviations and features frequent use of synonyms and homonyms, as well as the need to use and link multiple expert-curated ontologies and insufficient labeled data sets for the underlying NLP tools used [16-18]. The best previous embeddings, Embedding of Semantic Predications (ESP; detailed in the section *ESP Method*), which was designed for such literature-derived graphs, does not account for such *noise* due to NLP inference.

NLP algorithms quantify the confidence in their inference of extracted concepts and relations from the literature. Our hypothesis, which motivated this work, is that by using such confidence scores during representation learning, the learned embeddings would yield better features for predictive models. In this study, we developed techniques to use these confidence scores during representation learning to model inaccuracies in literature-derived KGs due to NLP inference. We illustrate the use of our technique on two well-known representation learning methods: DeepWalk [19] and Translating Embeddings for Modeling Multi-relational Data (TransE) [20]. We show how confidence scores can easily be incorporated in both these methods as *weights* that bias the methods to choose higher confidence edges and nodes over lower confidence edges and nodes during representation learning. Thus, we developed the *weighted* versions of these methods: *Weighted DeepWalk* and *Weighted TransE*.

We rigorously evaluated these methods on benchmark data sets for drug-ADE prediction and polypharmacy prediction. In both tasks, our weighted versions were able to learn representations that yielded more accurate predictive models than ESP and the unweighted versions of DeepWalk and TransE, with improvements of up to 5.75% in the F_1_-score and 8.4% in the area under the receiver operating characteristic curve (AUC) value. Thus, our experimental results demonstrate the benefit of modeling inaccuracies in the inferred KGs for representation learning. Better representations, in turn, lead to better classification models for ADE prediction.

### Background and Related Work

#### Biomedical KGs

The primary source of scientific clinical knowledge is biomedical literature, which records details of clinical trials conducted, case studies, and systematic reviews. To facilitate knowledge discovery from such unstructured data, standardized vocabularies and ontologies have been created; for example, the Unified Medical Language System Metathesaurus [21] contains more than 5 million clinical concepts—identified by controlled unique identifiers—that have been organized into structured ontologies.

NLP techniques that have been designed to infer both clinical concepts and their relations found in the literature automatically create ontologies from the rapidly growing body of biomedical literature. Data sources such as molecular databases, drug banks, or social media may also be used as additional inputs. Examples include the Semantic MEDLINE Database (SemMedDB) [22] and KnowLife [23]. These automatically generated ontologies have been found to be immensely useful to support hypothesis generation [12], literature-based knowledge discovery [24], and predictive modeling [25].

In this work, we used the SemMedDB, where clinical concepts are identified in PubMed abstracts through entity recognition algorithms and then mapped to their controlled unique identifiers. Various heuristics are used to infer the relations between concepts (see the study by Rindflesch and Fiszman [18] for details). The SemMedDB infers 30 different kinds of relations such as *Treats*, *Causes*, *Predisposes*, and *Prevents* among clinical concepts of various types that include diseases, drugs, procedures, and biological structures. These relations are organized into [*subject-predicate-object*] triplets (eg, [drug A-Treats-disease B]), where both the subject and object are clinical concepts and the predicate is a relation. There are more than 96 million such triplets extracted in the SemMedDB.

The SemMedDB contains useful information about each triplet including the following:

1. Co-occurrence scores of [subject-predicate-object] triplet: the number of times the triplet is inferred from the literature—higher number indicates higher confidence in the association.

(2) Subject-score and object-score: confidence score of the mapping found by NLP recognition algorithms between a text string and the subject or object concept. These scores were used in the methods we developed.

The collection of such triplets can be viewed as a *heterogeneous graph* comprising multiple vertex types (clinical concepts) and multiple edge types (predicates). This graph-based view enabled us to mine the KG using graph analytics tools. For instance, predicates only show direct relations between two concepts, whereas the graph illuminates indirect relations through various paths connecting the two concepts.

### Learning Clinical Concept Representations From KGs

#### Graph Embeddings

Statistical machine learning models typically assume inputs as feature vectors. To obviate the need for manual extraction of features from text and graph inputs, representation learning aims to learn features or representations from the input directly, in an unsupervised manner. Representation learning from graphs is an active research area; see the studies by Goyal and Ferrara [26] and Yang et al [27] for general surveys and the study by Wang et al [28] for a survey on representation learning on KGs. The representations are vectorial representations of the vertices of the graph. They are also called graph *embeddings* because of the similarities in both the representation learning algorithms and their uses to the word embeddings in NLP.

Formally, for any given graph *G = (V, E),* with vertex set *V* and edge set *E*, a graph representation learning algorithm learns a *d*-dimensional latent representation *x_vi_* ∈ 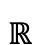
^d^, *d* « |*V*| for each vertex *v_i_* ∈ *V* that captures global structural and semantic relations (as described below) in the graph. We first outline skip-gram negative sampling (SGNS), a widely used neural architecture for obtaining word embeddings in NLP, and later describe how SGNS is used or adapted to obtain graph embeddings.

#### SGNS Approach

**Figure 2:**
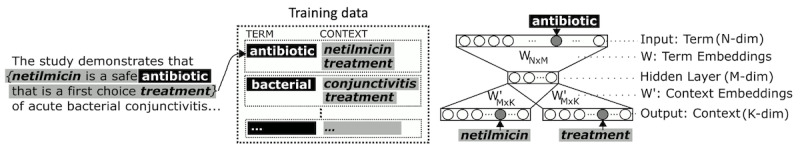
Skip-gram negative sampling: A window of words around a term constitutes its context. Word embeddings are obtained from the weights of a neural network trained to predict context words of a term. K-dim: K dimensions; M-dim: M dimensions; N-dim: N dimensions; SGNS: skip-gram negative sampling.

SGNS is a neural approach to learn word representations from text data [29]. The key idea is to train a neural network to predict the *context* of each word in the input text corpus, where context is defined as a window of neighboring words. Usually, preprocessing steps remove uninformative words such as stop-words (*a*, *an*, *the...*) before training, and one-hot encoding is used for input and output of the network. The window size is a parameter set during training. For each word, context words for every occurrence in the text corpus are extracted to form the training data (Figure 2). After the network is trained using gradient descent, the learned weights are used as word embeddings. The model can use negative samples—where words not found in the neighborhood are used—during training.

#### ESP Method

**Figure 3:**
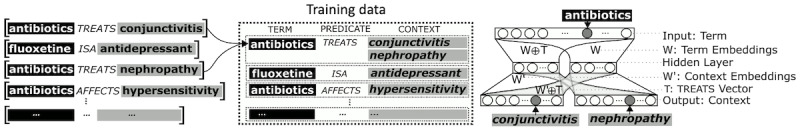
Embedding of Semantic Predications embeddings from [subject-predicate-object] triplets: the objects of a subject term form its context, and the skip-gram negative sampling architecture is modified to predict each context from the term and the predicate. ISA: is a.

To obtain embeddings of clinical concepts using [subject-predicate-object] triplets, also called predications, Cohen and Widdows [30] designed the ESP method based on the SGNS architecture. In ESP, the context of a *subject* concept is defined as the set of *objects* that it relates to through one or more predicates. In addition, their model was explicitly trained to enable analogical reasoning, with respect to biomedical relations, by defining binding operators (eg, exclusive OR [XOR], denoted by ⊕) on the representations of concepts and predicates. Thus, if there is a predicate such as drug A-Treats-disease B, then from the corresponding representations, they aim to have *drug A*⊕*treats≈disease B*. This is learned during training by modifying the SGNS architecture to predict the object (eg, disease B) from the XOR of the predicate and subject (drug A⊕treats; Figure 3). With these modifications, ESP obtains embeddings of both clinical concepts and predicates using a gradient descent–based optimization similar to that of SGNS.

ESP has not been developed by viewing the collection of triplets as a KG. When viewed from the KG perspective, we recognize that ESP trains its embeddings by using only its immediate neighbors in the graph. It is possible to learn embeddings that can explicitly incorporate more distant information, that is, by using a context for training that includes not just neighboring vertices but also vertices that are two or more hops away on the KG, for instance, through walk-based approaches that we describe next.

#### DeepWalk Method

**Figure 4:**
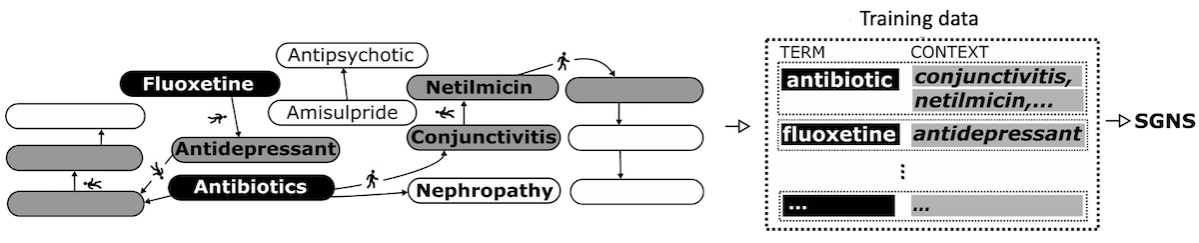
DeepWalk: Random walks generate contexts for a vertex, which are used as training data in skip-gram negative sampling to obtain embeddings for each vertex. SGNS: skip-gram negative sampling.

There are many graph embedding algorithms based on random walks. Although the details differ, they share the underlying idea of using random walks on the graph to define a context for a vertex and to generate training data similar to those for learning word embeddings. Then a neural architecture can be used to obtain vertex representations. We outline DeepWalk [19], one such walk-based algorithm. DeepWalk obtains training data through random walks from each vertex on the input graph and uses SGNS to obtain vertex representations (Figure 4). Note that DeepWalk assumes a homogeneous input graph; information regarding multiple vertex types and edge labels is not used and, hence, is not shown in Figure 4. The random walk generator randomly selects the next vertex to walk to from its neighborhood, that is, vertices that are connected by an edge. For each vertex in the input graph, select *N* sequences of *L* vertices each. In each sequence, at the kth step, the k+1st vertex is randomly sampled with probability:




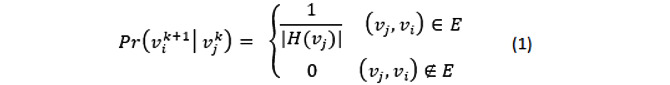




where *H*(*v_j_*) denotes the neighborhood of *v_j_*. Additional implementation details can be found in the study by Perozzi et al [19].

The simple idea of DeepWalk has been extended in many ways for homogeneous graphs. Relatively fewer walk-based approaches have been developed for heterogeneous graphs. Among them, Metapath2Vec is an effective method that uses *metapath schemes* to predefine the types of edges to be selected during random walk selection [31], an approach that works well in sparse graphs with relatively few edge types. However, the generation of such metapath schemes is difficult for biomedical KGs that are typically very dense and have many edge types. There are approaches, which are different from walk-based approaches, that have been designed directly for KGs, such as TransE, which we describe next.

#### TransE Method

**Figure 5:**
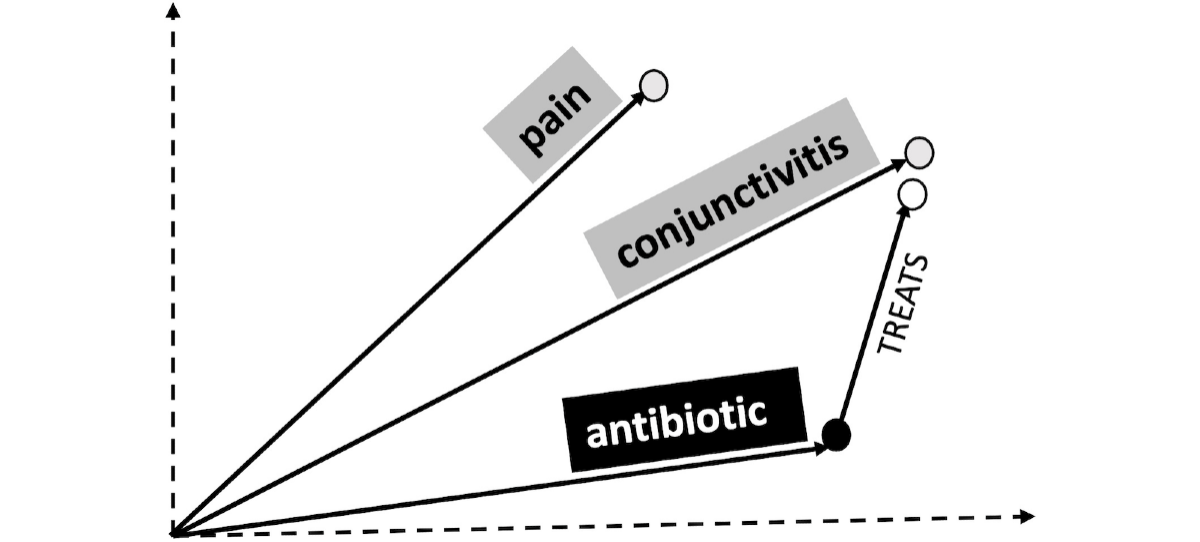
Schematic of TransE: Triplet (antibiotic, treats, conjunctivitis) is preserved in the vector sum of their representations in 2 dimensions: h[antibiotic] + l[treats] ~ t[conjunctivitis]. The vector sum h + l is much further from the vector for pain than from the vector for conjunctivitis. TransE: Translating Embeddings for Modeling Multi-relational Data.

We briefly describe the intuition behind TransE and refer the reader to the study by Bordes et al [20] for more details. For a set of clinical entities *E*, given a training set *S* of triplets (*h, l, t*)—composed of two clinical entities head *h* and tail *t* where *h*, *t* ∈ E—and a relationship (or predication) *l*, the TransE model learns *k*-dimensional vector representations of the entities and the relationships (where *k* is a hyperparameter). The idea behind the TransE model is that the relationship induced by the *l*-labeled edges corresponds to a translation of the vector representations. That is, we want that the vectors obey *h* + *l* ~ *t* when the predicate (*h*, *l*, *t*) is present in the KG. If the triplet (*h*, *l*, *t*) is not present, then the vector *h* + *l* should be far away from the tail concept *t* in vector space (Figure 5).

Note that the input of TransE is similar to that of ESP: both use [subject-predicate-object] triplets. However, ESP generates binary representations and has a different scheme for the composition of representations, whereas TransE obtains real-valued distributed representations with the usual operations defined on the vector space. Furthermore, TransE does not use SGNS for training and has a different energy-based framework for optimization as described below.

Following an energy-based framework, the energy of a triplet is given by *d*(*h* + *l*, *t*), where *d* is a dissimilarity function (eg, L1 or L2 norm); lower energy triplets are preferred because they preserve the required vector relationship. Therefore, to learn the vector representations, a margin-based ranking criterion, given below, is minimized over the set of triplets in the KG:




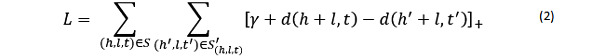




where [*x*]_+_ denotes the positive part of *x* and *y*>0 is a margin hyperparameter and *S'_(h,l,t)_* = {(*h'*, *l*, *t*)|*h'* ∈ *E*} ∪ {(*h*, *l*, *t'*)|*t'* ∈ *E*} is the set of corrupted triplets. The set *S'* is composed of training triplets with either the head diseases or tail diseases replaced by a random entity (but not both at the same time). The loss function *L* favors lower values of the energy for training triplets than for corrupted triplets and is thus a natural implementation of the intended criterion. The model uses stochastic gradient descent optimization to minimize the loss.

## Methods

### Model 1: Extending DeepWalk to Weighted DeepWalk

We made two modifications to DeepWalk. The first modification enabled us to sample edges in a heterogeneous graph without requiring fixed predefined metapath schemes. We then introduced a bias over the walks that was informed by simple statistics of the inferred clinical concepts and relations and, thus, accounted for inaccuracies during NLP inference. Both these approaches change the sampling strategy in the procedure for generating random walks of DeepWalk. Other steps involving SGNS for training remain the same.

We modified the random walk procedure such that an (edge, vertex) pair was selected for traversal at each step instead of just a vertex. Thus, if the same vertex could be reached through two different predicates (edges), they were considered two separate neighbors during the next step selection in the random walk. We viewed a subject vertex as one that was connected to not just another vertex, but also to a pair (predicate, object). Formally, we defined the set *E'* = {((*p*, *o*), *s*) iff [*s*, *p*, *o*] is a valid triplet}. This defines the neighborhood of a vertex *v_j_* as a set of (predicate, vertex) pairs: *H'*(*v_j_*) = {(*p*, *v_i_*)|((*p*, *v_i_*), *v_j_*) ∈ *E'*}*.* We incorporated information on the confidence scores from the SemMedDB using a scoring function in the sampling distribution at each step of the walk. As a result, the walks were biased toward vertices and edges with higher confidence. The selection of the next (edge, vertex) pair was performed by sampling from the distribution:




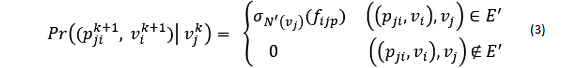




where *f*_ijp_ is a score for the corresponding triplet and σ*_N'(vj)_* is a softmax function over all the predicates from vertex *v_j_*. The triplet score was computed as a weighted product, 
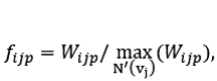
 where *W_ijp_* = (*w_j_s_vj_* × *w_i_s_vi_* × *w_p_c_p_*) and *s_v_* represents the score for the (subject and object) vertices and *c_p_* represents the co-occurrence score of the predicate.

The normalization, using the maximum value, was carried out to avoid numerical errors due to very large numbers. Each score had a multiplicative effect that resulted in triplets, with all 3 high scores being highly favored (for the next vertex selection in a walk) over triplets with any of the 3 scores being low. The weights were optimized through hyperparameter search. The softmax function was used to convert the scores to probabilities at each step of the walk. We performed L2 normalization on the learned representations to ensure that their L2 norms were equal to 1. We implemented both the random walk generators in Python (Python Software Foundation) and used the script from the study by Mikolov et al [29] for SGNS.

### Model 2: Extending TransE to Weighted TransE

Incorporating confidence scores in TransE is relatively straightforward. As described earlier, the loss function is designed in such a way that true triplets have lower energy than corrupted triplets. Using the weight function of the subject, object, and co-occurrence scores—*f_ijp_*, defined earlier for Weighted DeepWalk—we can simply reweight the energy of the true triplets in such a way that the higher-confidence triplets have lower energy than the lower-confidence triplets. As *f_ijp_* lies between 0 and 1, we can divide the energy of the true triplets to achieve this reweighting. Thus, the modified loss function becomes




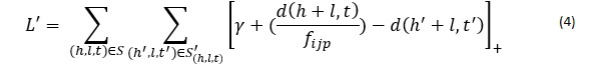




where [*x*]_+_ denotes the positive part of *x* and *y*>0 is a margin hyperparameter and *S'_(h,l,t)_* = {(*h'*, *l*, *t*)|*h'* ∈ *E*} ∪ {(*h*, *l*, *t'*)|*t'* ∈ *E*} is the set of corrupted triplets as described above.

### Drug-ADE Prediction

**Figure 6:**
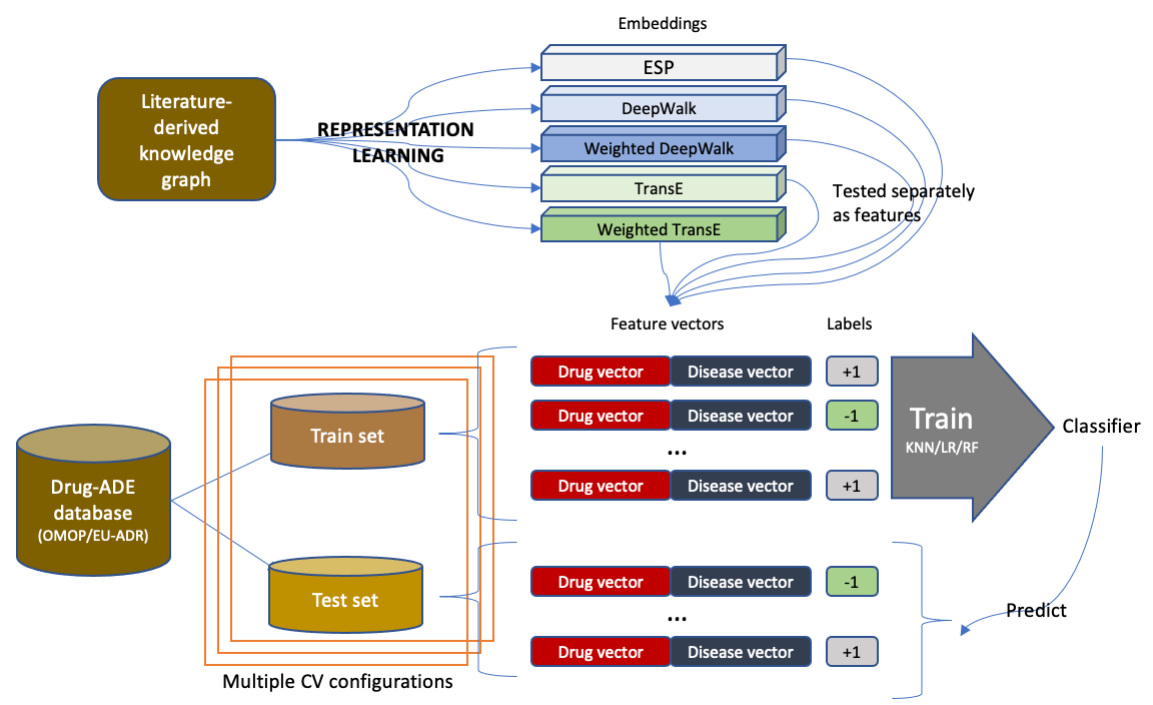
Experiment setting for drug–adverse drug reaction prediction. ADE: adverse drug reaction; CV: cross-validation; ESP: Embedding of Semantic Predications; EU-ADR: Exploring and Understanding Adverse Drug Reactions; KNN: k-nearest neighbors; LR: logistic regression; OMOP: Observational Medical Outcomes Partnership; RF: random forest; TransE: Translating Embeddings for Modeling Multi-relational Data.

Our first set of experiments followed the procedure described in the study by Mower et al [15] for ADE prediction. Figure 6 shows a schematic of the experiment setting, with details described in the following.

#### Data

We used 2 curated reference data sets that contain drug, disease pairs: Observational Medical Outcomes Partnership (OMOP) [32] and Exploring and Understanding Adverse Drug Reactions (EU-ADR) [8]. OMOP contains 4 ADEs: myocardial infarction, gastrointestinal bleeding, liver injury, and kidney injury for 180 drugs. The drugs for which embeddings from the SemMedDB could not be obtained were removed: 5 in OMOP (corresponding to the drugs darunavir and sitagliptin) and 1 in EU-ADR (for the drug nimesulide). Statistics of both the data sets used in our experiments are presented in Table 1.

ESP embeddings have been empirically evaluated for ADE prediction on these data sets. For each drug and ADE pair, a composite feature vector was obtained by binding the corresponding ESP embeddings. The use of these feature vectors in a logistic regression (LR) classifier was found to outperform previous literature-based methods [15].

**Table 1:** Exploring and Understanding Adverse Drug Reactions (EU-ADR) and Observational Medical Outcomes Partnership (OMOP) data set statistics.

Data set	Drugs	Diseases	ADE^a^ pairs	Non-ADE pairs
EU-ADR	65	10	43	50
OMOP	180	4	164	230

^a^ADE: adverse drug event.

#### Unsupervised Representation Learning

To compare the performance of the representation learning methods, we generated embeddings of all clinical concepts (nodes) in the SemMedDB using DeepWalk, TransE, Weighted DeepWalk, Weighted TransE, and ESP. We used the available implementation of DeepWalk [33] and TransE [34]. We experimented with multiple hyperparameter sets and selected the ones that yielded the best loss value during representation learning. In Weighted TransE and TransE, we set the α value to .001, batch size to 256 triplets, epochs to 100, and the number of corrupted triplets for each positive triplet to 1. The embedding dimension was 100 in both these models. For the DeepWalk and Weighted DeepWalk models, we set the walk length to 500, the number of walks to 20, window size to 4, α value to .025, and an embedding size of 256. For ESP, we used the embeddings provided by the authors [35], which had a dimension of 8000.

#### Classification Task and Algorithms

We used supervised binary classification to classify relationships consisting of (drug, disease) pairs. If a drug could cause a particular disease as a side effect, the label assigned to the pair was positive (+1); otherwise, the label assigned was negative (–1). In ESP, the XOR operator is used on each (drug, disease) pair’s vector representations (embeddings) to form a single 8000-dimensional input feature vector, which has also been provided by the authors [35]. For all the other embedding algorithms, for each (drug-disease) pair, we concatenated the vector representations (embeddings) learned from the SemMedDB KG of the drug and disease to form the input feature vectors. The performance of each representation learning technique was evaluated by their classification performance in this task.

To evaluate the downstream effect of the learned representations on classification performance, we used 3 different classifiers. LR is a commonly used linear model. For nonlinear classifiers, we used two different techniques: k-nearest neighbors (KNN) and random forest (RF). KNN classifies a test feature vector based on its distance from the k-nearest training data vectors. RF is an ensemble-based technique that uses multiple decision trees to make predictions. All the experiments in this study were conducted using the scikit-learn library (version 0.24.2) [36]. L1 regularization was used for the LR, information gain (entropy) criterion was used for the RF, and k=5 neighbors was used for the KNN classifier. All other parameters were retained at their default values.

#### Evaluation

Following the study by Mower et al [15], we evaluated the classifiers using leave-one-out (LOO) cross-validation and stratified 5-fold (S5F) cross-validation on the following data sets:

1. EU-ADR
2. OMOP
3. Combined EU-ADR+OMOP

In LOO cross-validation, the number of folds is equal to the number of instances in the data; in each fold, there is a single test instance, and the remaining instances are used as training data for the classifier. S5F cross-validation is an extension of regular 5-fold cross-validation, where the folds are made by preserving the percentage of samples for each class. In addition, we used two other settings (for a total of 5 settings) to evaluate the generalization performance:

- The classifiers were trained on EU-ADR data and tested on OMOP data.
- The classifiers were trained on OMOP data and tested on EU-ADR data.

Standard metrics to evaluate binary classification were used: the F_1_-score and the AUC value. Averages (over all the folds in case of S5F) are reported along with the SD. In the last two settings, we evaluated the trained model on the same 5 folds used in settings 1 and 2, respectively, to have performance values that could be compared.

### Visualization of Embeddings

To visually inspect the embeddings, we used the dimensionality reduction technique: t-distributed stochastic neighbor embedding (t-SNE) [37]. Embeddings from all 5 methods—for all 487 (drug, disease) pairs in both the OMOP and EU-ADR reference data sets—were plotted in 2 dimensions. The implementation used was from the sklearn library [36]. t-SNE was run with a learning rate of 600. To select the perplexity parameter, we empirically evaluated randomly chosen values and selected those that yielded the best cluster visualization. Perplexity was set to 30 to visualize ESP embeddings and to 5 for all other embeddings. In our plots, we represented the false drug-ADE pairs with an *o* sign and the true drug-ADE pairs with an *x* sign. Each disease was represented by a different color, and there were 10 diseases in total in the data sets.

### Polypharmacy Prediction

**Figure 7:**
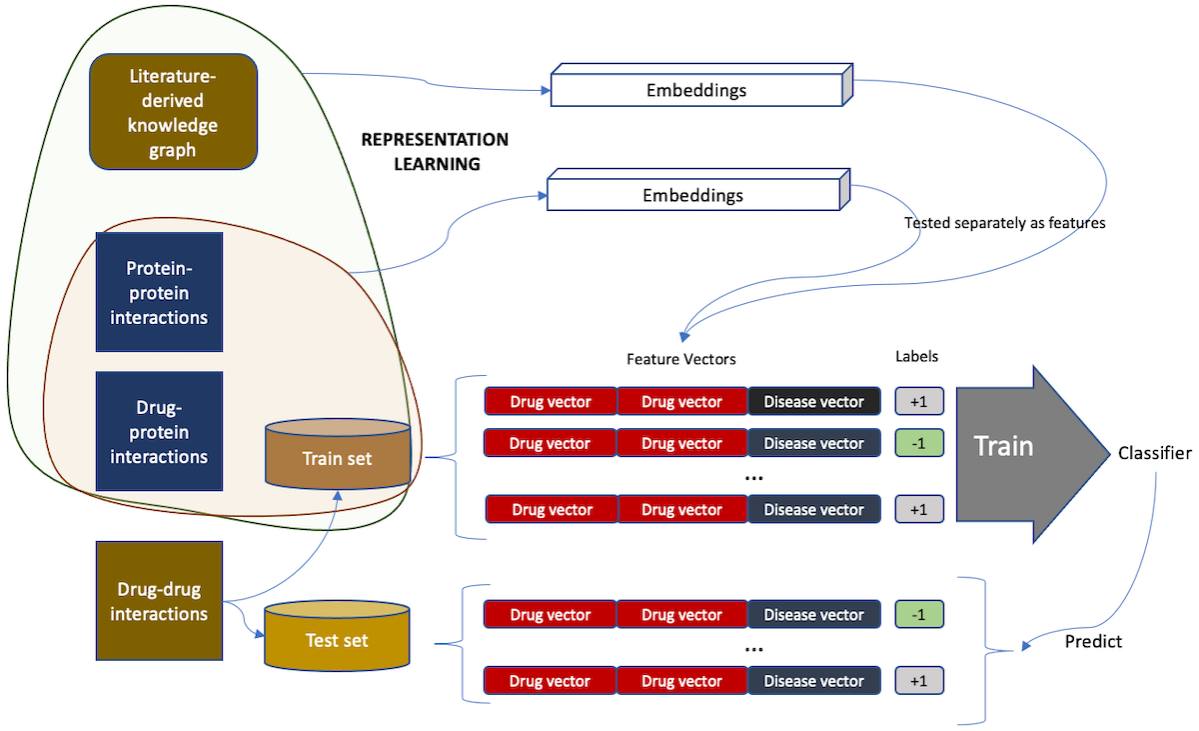
Experiment setting for polypharmacy prediction.

In this experiment, we evaluated the efficacy of our representation learning methods for polypharmacy prediction. Figure 7 shows a schematic of the experiment setting with details described in the following.

#### Data

We used the benchmark data set for polypharmacy prediction from the study by Zitnik et al [38], which was also used in the study by Burkhardt et al [39]. We used the same data set, including the exact train-test splits, and called it the polypharmacy data set.

The data consist of 3 types of interactions: drug-drug, drug-protein, and protein-protein interactions. Drug-drug interactions contain triplets of the form drug A-SE-drug B where consuming drug A and drug B together would cause the side effect (SE) mentioned in the triplet, for example, aspirin-Kidney-Failure-warfarin. These data were curated from two databases: Side Effect Resource (SIDER) [40] and Twosides [6]. We used the preprocessed data of the study by Burkhardt et al [39], downloaded from the Zenodo website [41], where triplets for side effects that occurred in fewer than 500 drug interactions are not used. There are 963 side effects in total. Protein-protein interactions, which were curated from multiple databases, indicate physical interactions that have been experimentally found in humans. Drug-protein interactions contain experimentally verified small chemicals (drugs) that target specific proteins. More details can be found in the study by Zitnik et al [38].

The drug-drug interaction data were divided into 80% training, 10% validation, and 10% test sets. Furthermore, along with valid interactions, that is, where the 2 drugs cause the reported side effect, the study by Zitnik et al [38] also provides an equal number of invalid interactions by using randomly selected drugs and side effects that do not occur in the valid interactions. There are 22,89,960 protein-protein interactions and 29,756 drug-protein interactions that were used only during training. Textbox 1 shows the number of interactions provided in the benchmark data set.

**Polypharmacy data set statistics.**

**Number of interactions provided in the benchmark data set**

- Train
  Drug-drug interactions: 73,23,790
  Protein-protein interactions (only used during training): 22,89,960
  Drug-protein interactions (only used during training): 29,756
- Test
  Valid drug-drug interactions (label 1): 4,57,196
  Invalid drug-drug interactions (label 0): 4,57,196

The previous best results published on this data set, to our knowledge, are those of ESP in the study by Burkhardt et al [39]. The authors’ approach first learns ESP embeddings from drug-drug, drug-protein, and protein-protein interactions of the polypharmacy training data set. Given a test triple—drug 1, drug 2, and side effect—they bind the embeddings of the drugs, and if the similarity of the obtained composite vector to the side effect embedding is more than a fixed threshold, they predict the triple to be valid. This simple approach was found to outperform Decagon, a more complex graph neural network–based approach [38].

#### Unsupervised Representation Learning

We generated embeddings in two different ways. First, we used the training data provided to learn embeddings using TransE, Weighted TransE, DeepWalk, and Weighted DeepWalk. We used the available implementations of DeepWalk [33] and TransE [34]. We experimented with multiple hyperparameter sets and selected the ones that yielded the best loss value during representation learning. Both DeepWalk and Weighted DeepWalk embeddings were generated by setting the number of walks to 25, walk length to 500, window size to 10, embedding size to 256, and α value to .025. TransE and Weighted TransE embeddings were generated by setting batch size to 512, number of corrupted triplets for each positive triplet to 1, epochs to 100, α value to .001, and embedding size to 100. In the weighted versions, the occurrence score for a triplet drug A-SE-drug B is the number of triplets containing the same drug A (subject) and drug B (object) with no restrictions on the side effect (ie, they may have different side effects), and subject, object scores were set to 1.

Second, to evaluate the utility of the SemMedDB as another auxiliary data source (in addition to protein-protein interaction and drug-protein interaction networks), we augmented the training data with 93,974,376 triplets from the SemMedDB. The subject, object scores and co-occurrence scores of the SemMedDB were reused as such for the SemMedDB; for the polypharmacy data set, the subject, object scores and co-occurrence scores were set to the highest values found in the SemMedDB (which were 1000, 1000, and 33,478, respectively). As this was a much larger graph, different hyperparameters were used to obtain embeddings. DeepWalk embeddings were generated by setting the number of walks to 375, walk length to 500, window size to 10, embedding size to 256, and α value to .025. TransE and Weighted TransE embeddings were generated by setting batch size to 512, number of corrupted triplets for each positive triplet to 2, epochs to 1500, α value to .001, and embedding size to 100.

#### Classification Task and Settings

The binary classification task was to distinguish the valid and invalid drug-drug interactions (in the test set provided). After the embeddings were learned (separately in the two settings), we trained an RF classifier from sklearn—with the number of decision trees set to 100 and maximum depth of each tree set to 20 (all other settings were unchanged from the default)—for this task. For each drug-SE-drug interaction, the embeddings of both the drugs and the side effect were concatenated and used as a feature vector in the classifier. This concatenation yielded a 300-dimensional feature vector for each triplet in the case of TransE and Weighted TransE and a 768-dimensional feature vector in the case of DeepWalk and Weighted DeepWalk.

As there were no invalid interactions in the training set, we randomly generated 73,23,790 pairs of drug-drug interactions such that they did not occur in either the training set or test set provided in the benchmark data.

#### Evaluation Metrics

The evaluation metrics used were the same as the ones used in the studies by Zitnik et al [38] and Burkhardt et al [39]: AUC, area under the precision-recall curve (AUPRC), and average precision at 50 (AP@50) for each of the 963 side effects, which were then averaged. We compared our results with the published results reported on the same data set in the study by Burkhardt et al [39], which used ESP-based embeddings, and in the study by Zitnik et al [38], which used Decagon, a graph convolutional network developed for this task.

## Results

### Drug-ADE Prediction

Tables 2-4 show the F_1_-scores on the LOO and S5F cross-validation configurations obtained by the algorithms for the data sets OMOP, EU-ADR, and the combined OMOP+EU-ADR data set, respectively. In most cases, we observed that ESP outperformed TransE and DeepWalk. However, both the weighted versions—Weighted TransE and Weighted DeepWalk—outperformed ESP in most cases. Among the 3 classifiers, for the same embeddings, RF outperformed LR and KNN in most cases. Overall, Weighted TransE with RF had the best performance in most cases, with improvements of up to 5.75% over ESP.

**Table 2:** F_1_-scores from leave-one-out (LOO) and stratified 5-fold (S5F) cross-validation (CV) configurations on the Observational Medical Outcomes Partnership data set.

Model	ESP^a^	TransE^b^	DeepWalk	Weighted DeepWalk	Weighted TransE	Increase (%)^c^
LR^d^ S5F, mean (SD)	0.895 (0.02)	0.861 (0.0185)	0.813 (0.024)	0.899 (0.0186)	0.915^e^ (0.0178)	2.23
LR LOO-CV	0.901	0.889	0.828	0.912	0.923^e^	2.44
KNN^f^ S5F, mean (SD)	0.816 (0.016)	0.793 (0.0163)	0.784 (0.0173)	0.814 (0.0167)	0.838^e^ (0.0155)	2.69
KNN LOO-CV	0.837	0.804	0.796	0.837	0.859^e^	2.63
RF^g^ S5F, mean (SD)	0.906 (0.008)	0.865 (0.0078)	0.82 (0.0091)	0.91 (0.0077)	0.923^e^ (0.0069)	1.87
RF LOO-CV	0.921	0.877	0.834	0.931	0.936^e^	1.69

^a^ESP: Embedding of Semantic Predications.

^b^TransE: Translating Embeddings for Modeling Multi-relational Data.

^c^Improvement in performance of the best method over Embedding of Semantic Predications (in each row).

^d^LR: logistic regression.

^e^Best result in each row.

^f^KNN: k-nearest neighbors.

^g^RF: random forest.

**Table 3:** F_1_-scores from leave-one-out (LOO) and stratified 5-fold (S5F) cross-validation (CV) configurations on the Exploring and Understanding Adverse Drug Reactions data set.

Model	ESP^a^	TransE^b^	DeepWalk	Weighted DeepWalk	Weighted TransE	Increase (%)^c^
LR^d^ S5F, mean (SD)	0.834 (0.066)	0.823 (0.073)	0.769 (0.089)	0.832 (0.068)	0.857^e^ (0.0635)	2.76
LR LOO-CV	0.841	0.827	0.783	0.843	0.864^e^	2.73
KNN^f^ S5F, mean (SD)	0.621 (0.085)	0.55 (0.096)	0.646^e^ (0.076)	0.639 (0.079)	0.643 (0.0732)	4.02
KNN LOO-CV	0.641	0.651	0.659	0.665^e^	0.663	3.74
RF^g^ S5F, mean (SD)	0.835 (0.017)	0.824 (0.022)	0.77 (0.035)	0.856^e^ (0.0157)	0.855 (0.0162)	2.51
RF LOO-CV	0.85	0.826	0.785	0.874	0.877^e^	3.17

^a^ESP: Embedding of Semantic Predications.

^b^TransE: Translating Embeddings for Modeling Multi-relational Data.

^c^Improvement in performance of the best method over Embedding of Semantic Predications.

^d^LR: logistic regression.

^e^Best result in each row.

^f^KNN: k-nearest neighbors.

^g^RF: random forest.

**Table 4:** F_1_-scores from leave-one-out (LOO) and stratified 5-fold (S5F) cross-validation (CV) configurations on combined Observational Medical Outcomes Partnership+Exploring and Understanding Adverse Drug Reactions data set.

Model	ESP^a^	TransE^b^	DeepWalk	Weighted DeepWalk	Weighted TransE	Increase (%)^c^
LR^d^ S5F, mean (SD)	0.886 (0.021)	0.855 (0.033)	0.843 (0.039)	0.897 (0.025)	0.904^e^ (0.0203)	2.03
LR LOO-CV	0.911	0.871	0.856	0.928	0.934^e^	2.52
KNN^f^ S5F, mean (SD)	0.817 (0.035)	0.768 (0.043)	0.755 (0.049)	0.831 (0.047)	0.836^e^ (0.032)	2.32
KNN LOO-CV	0.829	0.79	0.776	0.842	0.848^e^	2.29
RF^g^ S5F, mean (SD)	0.86 (0.021)	0.86 (0.024)	0.845 (0.025)	0.892 (0.021)	0.898^e^ (0.0208)	4.42
RF LOO-CV	0.87	0.868	0.862	0.898	0.92^e^	5.75

^a^ESP: Embedding of Semantic Predications.

^b^TransE: Translating Embeddings for Modeling Multi-relational Data.

^c^Improvement in performance of the best method over Embedding of Semantic Predications.

^d^LR: logistic regression.

^e^Best result in each row.

^f^KNN: k-nearest neighbors.

^g^RF: random forest.

Tables 5-7 show the AUC values for the LOO and S5F cross-validation configurations obtained by the algorithms for the data sets OMOP, EU-ADR, and the combined OMOP+EU-ADR data set, respectively. We observed the same trend that was observed with the F_1_-scores. After accounting for the weights, the results for both DeepWalk and TransE improved over those of ESP. On the OMOP data set, LR with Weighted TransE obtained the highest improvement of 3.85%. On the EU-ADR data set, KNN performed the best and with both the weighted versions improved over ESP by approximately 8%. On the combined data set, KNN with Weighted TransE had the highest improvement over ESP.

**Table 5:** Area under the receiver operating characteristic curve values from leave-one-out (LOO) and stratified 5-fold (S5F) cross-validation (CV) configurations on the Observational Medical Outcomes Partnership data set.

Model	ESP^a^	TransE^b^	DeepWalk	Weighted DeepWalk	Weighted TransE	Increase (%)^c^
LR^d^ S5F, mean (SD)	0.935 (0.024)	0.935 (0.022)	0.892 (0.026)	0.932 (0.0287)	0.971^e^ (0.023)	3.85
LR LOO-CV	0.94	0.938	0.901	0.963	0.965^e^	2.66
KNN^f^ S5F, mean (SD)	0.883 (0.023)	0.858 (0.024)	0.841 (0.0267)	0.891 (0.021)	0.911^e^ (0.027)	3.17
KNN LOO-CV	0.902	0.875	0.857	0.894	0.924^e^	2.44
RF^g^ S5F, mean (SD)	0.945 (0.008)	0.931 (0.0091)	0.888 (0.0078)	0.958 (0.0077)	0.971^e^ (0.0069)	2.75
RF LOO-CV	0.961	0.943	0.881	0.971	0.972^e^	1.14

^a^ESP: Embedding of Semantic Predications.

^b^TransE: Translating Embeddings for Modeling Multi-relational Data.

^c^Improvement in performance of the best method over Embedding of Semantic Predications.

^d^LR: logistic regression.

^e^Best result in each row.

^f^KNN: k-nearest neighbors.

^g^RF: random forest.

**Table 6:** Area under the receiver operating characteristic curve values from leave-one-out (LOO) and stratified 5-fold (S5F) cross-validation (CV) configurations on the Exploring and Understanding Adverse Drug Reactions data set.

Model	ESP^a^	TransE^b^	DeepWalk	Weighted DeepWalk	Weighted TransE	Increase (%)^c^
LR^d^ S5F, mean (SD)	0.885 (0.085)	0.897 (0.076)	0.825 (0.0732)	0.901 (0.096)	0.929^e^ (0.0743)	4.97
LR LOO-CV	0.903	0.899	0.843	0.902	0.919^e^	1.77
KNN^f^ S5F, mean (SD)	0.693 (0.076)	0.634 (0.072)	0.753 (0.074)	0.702 (0.083)	0.712^e^ (0.087)	8.66
KNN LOO-CV	0.721	0.734	0.778	0.784^e^	0.752	8.74
RF^g^ S5F, mean (SD)	0.894 (0.0164)	0.897 (0.089)	0.826 (0.068)	0.924^e^ (0.0635)	0.924^e^ (0.066)	3.36
RF LOO-CV	0.922	0.901	0.847	0.927	0.933^e^	1.19

^a^ESP: Embedding of Semantic Predications.

^b^TransE: Translating Embeddings for Modeling Multi-relational Data.

^c^Improvement in performance of the best method over Embedding of Semantic Predications.

^d^LR: logistic regression.

^e^Best result in each row.

^f^KNN: k-nearest neighbors.

^g^RF: random forest.

**Table 7:** Area under the receiver operating characteristic curve values from leave-one-out (LOO) and stratified 5-fold (S5F) cross-validation (CV) configurations on the combined Observational Medical Outcomes Partnership+Exploring and Understanding Adverse Drug Reactions data set.

Model	ESP^a^	TransE^b^	DeepWalk	Weighted DeepWalk	Weighted TransE	Increase (%)^c^
LR^d^ S5F, mean (SD)	0.932 (0.027)	0.925 (0.021)	0.901 (0.026)	0.93 (0.024)	0.945^e^ (0.023)	1.39
LR LOO-CV	0.952	0.935	0.923	0.969	0.972^e^	2.1
KNN^f^ S5F, mean (SD)	0.885 (0.033)	0.823 (0.025)	0.806 (0.0203)	0.901 (0.0227)	0.906^e^ (0.039)	2.37
KNN LOO-CV	0.898	0.855	0.837	0.9	0.923^e^	2.78
RF^g^ S5F, mean (SD)	0.92 (0.038)	0.93 (0.042)	0.9 (0.025)	0.937 (0.0247)	0.94^e^ (0.026)	2.17
RF LOO-CV	0.951^e^	0.92	0.929	0.935	0.94	–1.1

^a^ESP: Embedding of Semantic Predications.

^b^TransE: Translating Embeddings for Modeling Multi-relational Data.

^c^Improvement in performance of the best method over Embedding of Semantic Predications.

^d^LR: logistic regression.

^e^Best result in each row.

^f^KNN: k-nearest neighbors.

^g^RF: random forest.

Table 8 shows the performance of the methods when the embeddings and classifiers were trained on OMOP data and prediction was carried out on EU-ADR data. Table 9 shows the results of the opposite case: training on EU-ADR data and prediction on OMOP data. Compared with the values in Tables 2-7, we observed lower F_1_-scores and AUC values in general. As the test set was from a different source, it was harder for the trained models to generalize. The performance trend across the algorithms remained the same: DeepWalk and TransE did not outperform ESP, but our weighted versions outperformed ESP. Weighted TransE had the best performance in most cases, with improvements of up to 8.4% in AUC value and 3.7% in the F_1_-score with LR (as seen in Table 9).

**Table 8:** Area under the receiver operating characteristic curve (AUC) values and F_1_-scores: training on Observational Medical Outcomes Partnership data and prediction on Exploring and Understanding Adverse Drug Reactions data.

Model and metric		ESP^a^, mean (SD)	TransE^b^, mean (SD)	DeepWalk, mean (SD)	Weighted DeepWalk, mean (SD)	Weighted TransE, mean (SD)	Increase (%)^c^
**Logistic regression**							
	F_1_	0.715 (0.023)	0.711 (0.026)	0.703 (0.031)	0.734 (0.037)	0.737^d^ (0.027)	3.07
	AUC	0.798 (0.017)	0.788 (0.023)	0.769 (0.0164)	0.803^d^ (0.018)	0.802 (0.019)	0.63
**KNN^e^**							
	F_1_	0.712 (0.028)	0.702 (0.038)	0.698 (0.042)	0.729 (0.029)	0.734^d^ (0.024)	3.09
	AUC	0.785 (0.022)	0.767 (0.021)	0.764 (0.023)	0.801 (0.019)	0.804^d^ (0.027)	2.42
**Random forest**							
	F_1_	0.724 (0.036)	0.714 (0.039)	0.710 (0.041)	0.745 (0.022)	0.748^d^ (0.021)	3.31
	AUC	0.815 (0.007)	0.800 (0.008)	0.783 (0.005)	0.818 (0.006)	0.825^d^ (0.007)	1.23

^a^ESP: Embedding of Semantic Predications.

^b^TransE: Translating Embeddings for Modeling Multi-relational Data.

^c^Improvement in performance of the best method over Embedding of Semantic Predications.

^d^Best result in each row.

^e^KNN: k-nearest neighbors.

**Table 9:** Area under the receiver operating characteristic curve (AUC) scores and F_1_-scores: training on Exploring and Understanding Adverse Drug Reactions data and prediction on Observational Medical Outcomes Partnership data.

Model and metric		ESP^a^, mean (SD)	TransE^b^, mean (SD)	DeepWalk, mean (SD)	Weighted DeepWalk, mean (SD)	Weighted TransE, mean (SD)	Increase (%)^c^
**Logistic regression**							
	F-1	0.612 (0.018)	0.604 (0.028)	0.597 (0.034)	0.632 (0.019)	0.635^d^ (0.021)	3.76
	AUC	0.680 (0.028)	0.678 (0.019)	0.666 (0.022)	0.739^d^ (0.025)	0.737 (0.021)	8.67
**KNN^e^**							
	F-1	0.609 (0.028)	0.598 (0.036)	0.589 (0.033)	0.628 (0.25)	0.633^d^ (0.0223)	3.94
	AUC	0.684 (0.004)	0.665 (0.008)	0.648 (0.003)	0.731 (0.006)	0.734^d^ (0.007)	7.31
**Random forest**							
	F-1	0.630 (0.032)	0.617 (0.035)	0.601 (0.043)	0.641 (0.029)	0.651^d^ (0.0286)	3.33
	AUC	0.717 (0.022)	0.685 (0.017)	0.675 (0.019)	0.750 (0.023)	0.763^d^ (0.027)	6.41

^a^ESP: Embedding of Semantic Predications.

^b^TransE: Translating Embeddings for Modeling Multi-relational Data.

^c^Improvement in performance of the best method over Embedding of Semantic Predications.

^d^Best result in each row.

^e^KNN: k-nearest neighbors.

### Visualization

**Figure 8:**
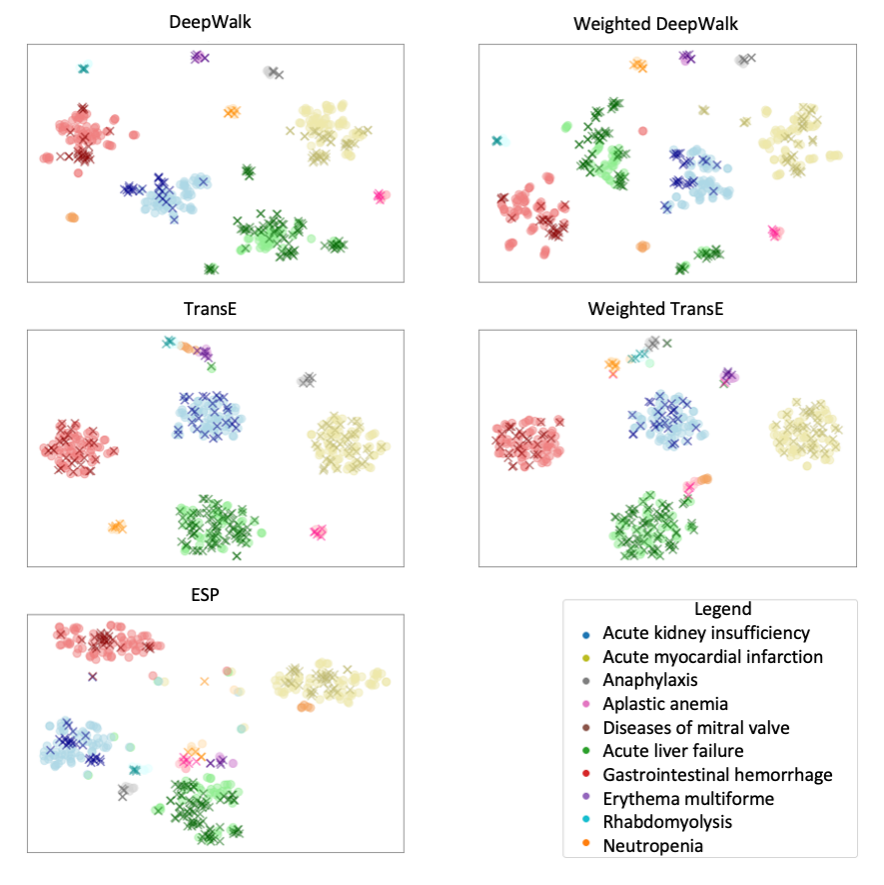
t-distributed stochastic neighbor embedding plots of disease-drug embeddings of the combined Exploring and Understanding Adverse Drug Reactions+Observational Medical Outcomes Partnership data set. Color indicates disease, and the markers x and o indicate presence and absence, respectively, of a side effect. ESP: Embedding of Semantic Predications; TransE: Translating Embeddings for Modeling Multi-relational Data.

Figure 8 shows the t-SNE visualizations of the embeddings obtained from each of the methods. The cluster structure with respect to the diseases is clear in all the embeddings. The intercluster separation seems to be the best for TransE and its weighted version, where the clusters (after the t-SNE dimensionality reduction) are also more compactly distributed.

Within each cluster, the positive and negative instances do not appear to be well separated, although there is some localization seen in the ESP, DeepWalk, and Weighted DeepWalk clusters. The results in the previous sections show that, among the classifiers we tested, RF and KNN had the best performance. Both indicated that the boundary between positive and negative instances was nonlinear.

### Polypharmacy Prediction

We first used TransE, DeepWalk, and their weighted versions to obtain embeddings from the data (without the use of the SemMedDB). As shown in Table 10, the performance of all 4 methods was superior to that of ESP in terms of the mean AUC value and mean AUPRC value. DeepWalk outperformed TransE. The weighted versions were not superior presumably because the underlying graph was not noisy and the weights based on co-occurrences alone in the drug-drug interaction graph did not affect the representation learning.

When the SemMedDB was added as an additional data source from which to learn embeddings, the results improved, as shown in Table 10. The maximum increase was seen in the AP@50 metric for both TransE and DeepWalk. This shows that the addition of the SemMedDB during representation learning improves the precision of the classifier learned. The weighted versions were not significantly better than the corresponding unweighted versions because the Decagon graphs had a significantly higher number of triplets than the SemMedDB, which dominated the data.

Overall, the representations learned with Weighted DeepWalk on the SemMedDB and polypharmacy graphs (drug-drug, drug-protein, and protein-protein interactions), when used in the RF classifier obtained the best results advancing the state of the art by 3.5% in the mean AUC value, 4.3% in the mean AUPRC value, and 5% in the mean AP@50 value.

**Table 10:** Mean (SD) area under the receiver operating characteristic curve (AUC), area under the precision-recall curve (AUPRC), and average precision at 50 (AP@50) values (averaged over 963 side effects) on the polypharmacy data set.

Metric	Polypharmacy graphs, mean (SD)					SemMedDB^a^+polypharmacy graphs, mean (SD)					Published results	
	TransE^b^	Weighted TransE	DeepWalk	Weighted DeepWalk	TransE		Weighted TransE	DeepWalk	Weighted DeepWalk	ESP^c^		Decagon
Mean AUC	0.921 (0.021)	0.921 (0.021)	0.932 (0.019)	0.932 (0.019)	0.926 (0.024)		0.924 (0.024)	0.935^d^ (0.020)	0.935^d^ (0.020)	0.903 (0.023)		0.872
Mean AUPRC	0.877 (0.037)	0.875 (0.038)	0.91 (0.030)	0.911 (0.030)	0.906 (0.033)		0.904 (0.033)	0.912 (0.031)	0.913^d^ (0.031)	0.875 (0.034)		0.832
Mean AP@50	0.73 (0.165)	0.725 (0.167)	0.896 (0.082)	0.896 (0.081)	0.915 (0.061)		0.916 (0.061)	0.906 (0.066)	0.909^d^ (0.064)	0.865 (0.073)		0.803

^a^SemMedDB: Semantic MEDLINE Database.

^b^TransE: Translating Embeddings for Modeling Multi-relational Data.

^c^ESP: Embedding of Semantic Predications.

^d^Best result in each row.

## Discussion

### Principal Findings

Unsupervised representation learning enables us to find useful features from data without requiring task-specific labels, which can subsequently be used in multiple applications. This is particularly useful when labeled data for a specific task are scarce, such as in ADE prediction, and when the data are complex, which is the case for KGs. Biomedical KGs such as the SemMedDB are inferred from the literature through NLP. This inference process introduces noise in the form of erroneous or incomplete edges and nodes in the KG. We developed new techniques to model underlying noise during representation learning from literature-derived biomedical KGs. During NLP inference, confidence scores were assigned to the inferred clinical concepts (vertices) and relations (edges). Our method effectively used these confidence scores during representation learning to model the inaccuracies in the graphs due to NLP inference.

We illustrated the use of our technique on two well-known representation learning methods: DeepWalk and TransE. We showed how confidence scores can easily be incorporated in both these methods to develop their *weighted* versions: Weighted DeepWalk and Weighted TransE. We compared the performance of these methods with ESP, which is, to our knowledge, the best-known representation learning method designed for the SemMedDB, a literature-derived KG. All the experiments were performed on benchmark data sets for ADE prediction.

In one set of experiments, the drug and disease embeddings learned from various representation learning methods were used to train classifiers and predict on held-out test sets in various cross-validation configurations. In another set of experiments, the side effects of drug-drug interactions were predicted using other drug-drug interactions as well as auxiliary data on drug-protein and protein-protein interactions. In the latter case, representations were learned both with and without the use of KGs. In both sets of experiments, our weighted versions learned representations that yielded more accurate predictive models than ESP as well as the unweighted versions of DeepWalk and TransE. Visual inspection of the learned embeddings shows a clear cluster structure in compressed 2-dimensional view, indicating that the disease and drug embeddings have been learned well from the KG.

In the second set of experiments, the use of biomedical KG as an auxiliary data source was found to considerably improve the precision. When the KG was not used as an auxiliary source, our weighted versions did not outperform the unweighted versions of DeepWalk and TransE for representation learning from drug-drug, drug-protein, and protein-protein interaction graphs. These graphs are not literature-derived, and the weights were based on co-occurrence scores in lieu of confidence scores. This shows that when the underlying graphs are not noisy, the weights may not add much value, although the performance does not deteriorate.

Our weighted versions of DeepWalk and TransE are, by design, biased toward triplets that have high co-occurrence scores in literature-derived KGs. This may not favor *some* relations that have low co-occurrence scores. The low score may be due to the triplet being a recently discovered relation or because it may be mentioned infrequently in the literature. However, the aim of graph representation learning methods is to use the entire KG, including indirectly related concepts, to learn the representation of a clinical concept. Therefore, if there are other (older or more frequent) relations that strongly indicate the possibility of the relation with low co-occurrence, then this signal is captured during representation learning. It is exactly this ability of graph representation learning that makes it useful in link prediction for knowledge discovery [20].

To evaluate this in our specific context, we checked the predictive accuracy of our weighted approach using the EU-ADR data set for those true drug-ADE pairs that have relations with low co-occurrence scores in the SemMedDB. Table 11 lists the predicates and their co-occurrence scores for 3 drug (subject) and disease (object) pairs. Note that these co-occurrence scores are much smaller than the maximum value of 33,478.

**Table 11:** Drug, adverse drug event (ADE) pairs from the Exploring and Understanding Adverse Drug Reactions data set with low co-occurrence scores in the Semantic MEDLINE Database.

Drug	ADE	Predicates (co-occurrence score)
Diclofenac	Anaphylaxis	Causes (6), Predisposes (1)
Aspirin	Anaphylaxis	Disrupts (2), Augments (2), Affects (2), Causes (7), Treats (1)
Acetaminophen	Anaphylaxis	Causes (7), Treats (3), Affects (1)

We checked the predictions for each of the aforementioned 3 pairs using classifiers trained on the SemMedDB representations generated using Weighted DeepWalk. In all, 3 classifiers—RF, KNN, and LR—were trained on EU-ADR data after excluding the pair being tested. The features were obtained by concatenating the corresponding drug and disease representations, as was done for the experiments on drug-ADE prediction. All 3 classifiers correctly identified the 3 pairs as true positives. This strongly suggests that the representations could learn the indirect relations from the KG despite being biased through our weighted approach toward relations with high co-occurrence scores.

To summarize, all our experimental results clearly highlight the importance of modeling inaccuracies in the inferred KGs for representation learning.

### Limitations

This study has the following limitations. Our weighting technique relies on the confidence scores provided and thus, in turn, depends on the accuracy of these scores. Errors in these scores may be detrimental to representation learning, and their effects need to be evaluated further. Model designers should be aware of this limitation when such weighting schemes are used in other KGs.

We evaluated the use of our weighting scheme on just two representation learning methods: DeepWalk and TransE. Many other methods exist, especially for heterogeneous networks; a recent survey can be found in the study by Yang et al [27]. Despite the simplicity of these approaches, we obtained very good results, outperforming the state of the art for ADE prediction. We believe that the underlying idea of our weighting scheme can be applied to many other representation learning methods, which can be investigated in the future.

In our experiments, both OMOP and EU-ADR data sets were not large. Although in our experiments, we rigorously tested many cross-validation configurations, accuracy values can differ in other data sets. We also note that the reported performance was dependent on the KG used to learn representations from, and the results may vary with other KGs. This is less of a concern for the second polypharmacy data set, which was much larger. The relative performances of the methods showed a consistent trend across both data sets. Comparisons with more diverse data sets will further our understanding of the strengths and limitations of these methods.

### Future Work

This work can be extended in many ways. Alternative approaches to designing the scoring function and weighting scheme used in our weighting function can be investigated. In large data sets, it may be possible to learn the weights automatically from the data by suitably modifying the models. The weighting scheme can be extended to incorporate additional information in literature-derived KGs. To leverage the underlying biomedical literature used, techniques to obtain causal assessment can also be explored.

Additional experiments can be designed to compare our approach with a fully supervised approach where both the embeddings and the classifier are learned jointly. Future work can also evaluate the effects of KG characteristics on the performance by experimenting with other KGs. Finally, the utility of our representations in other tasks such as diagnosis prediction or finding new clinical associations can also be evaluated.

### Conclusions

Literature-derived KGs are an important resource for analyzing the wealth of knowledge stored in the growing biomedical literature. These KGs are inferred through NLP techniques, and their limitations may result in incomplete or erroneous nodes and edges. KG embeddings provide a scalable and automatic way of obtaining features from KGs that can be valuable in multiple biomedical prediction tasks. Our work demonstrates the need for modeling noise in the underlying KG and makes an important step toward improved representation learning from literature-derived KGs and thus toward effectively using literature-derived KGs for predictive models.

Our experiments show that such *noise-aware* representations in turn lead to classifiers for ADE prediction that are more accurate than representations learned from the best previous methods. The new models in this work can be used by pharmacovigilance teams to detect previously unknown ADEs for further evaluation. Software implementation of our new methods and all experiments are publicly available at our website [42].

## Abbreviations

ADE: adverse drug event
AP@50: average precision at 50
AUC: area under the receiver operating characteristic curve
AUPRC: area under the precision-recall curve
ESP: Embedding of Semantic Predications
EU-ADR: Exploring and Understanding Adverse Drug Reactions
KG: knowledge graph
KNN: k-nearest neighbors
LOO: leave-one-out
LR: logistic regression
NLP: natural language processing
OMOP: Observational Medical Outcomes Partnership
RF: random forest
S5F: stratified 5-fold
SemMedDB: Semantic MEDLINE Database
SGNS: skip-gram negative sampling
SIDER: Side Effect Resource
TransE: Translating Embeddings for Modeling Multi-relational Data
t-SNE: t-distributed stochastic neighbor embedding
XOR: exclusive OR
